# Reassessing evidence for symmetric histone inheritance in *Drosophila* stem cells

**DOI:** 10.1073/pnas.2531792123

**Published:** 2026-03-06

**Authors:** Xin Chen

**Affiliations:** ^a^HHMI, Baltimore, MD 21218; ^b^Department of Biology, The Johns Hopkins University, Baltimore, MD 21218

Li et al. reevaluated histone inheritance in *Drosophila* stem cells using 1-2 tagged-*H3.1* among ~200 endogenous copies (with *cis*-acting sequence changes in the germline) (1). Here, we discuss their statistical analyses, experimental design, and data acquisition that we assert significantly compromise their conclusions.

**Fig. 1.**
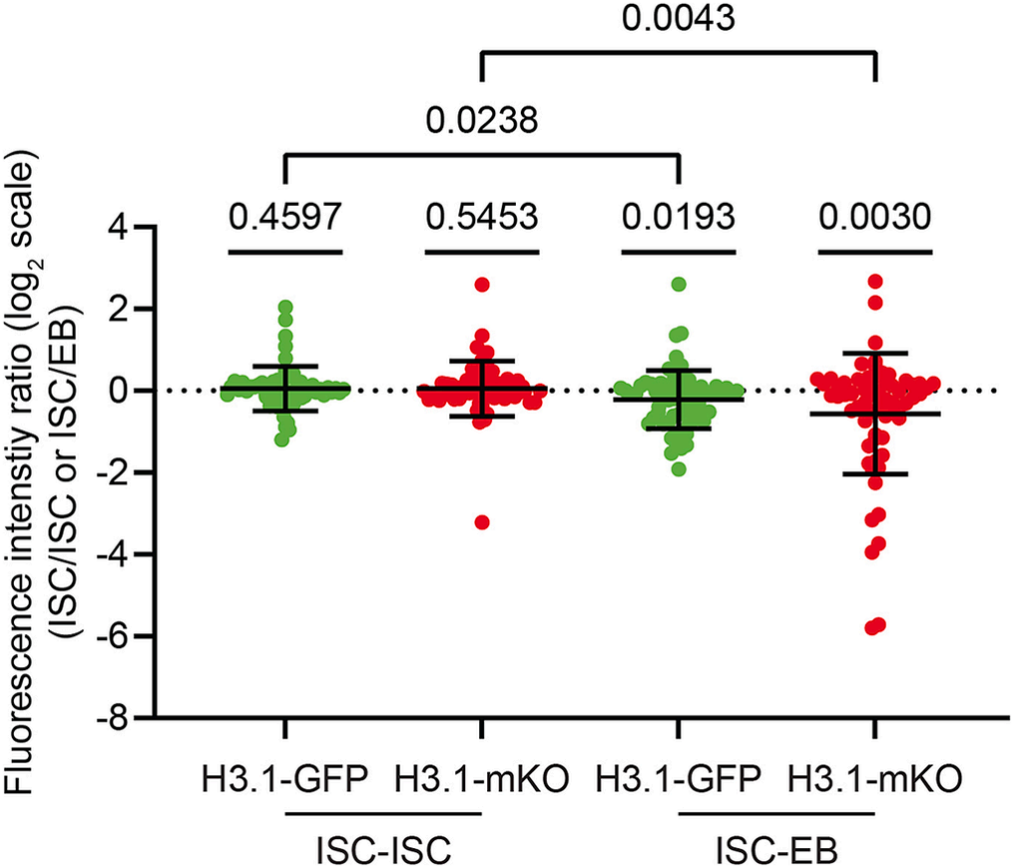
Reanalysis of H3.1-GFP (old) and H3.1-mKO (new) in ISC-ISC and ISC-EB pairs. Reanalysis of Li et al.’s *SI Appendix*, figure S8, on H3.1-GFP (old) and H3.1-mKO (new) distributions in ISC-EB and ISC-ISC pairs, reveals that both old and new H3.1 differ significantly from log_2_ = 0 in ISC-EB pairs (*P* < 0.05) but not in ISC-ISC pairs (*P* > 0.45). H3.1-GFP and H3.1-mKO ratios are compared against the null hypothesis (log_2_ = 0) with one sample *t* test. Pairwise comparisons of the same histone across division modes (old H3.1 in ISC-EB vs. ISC-ISC; new H3.1 in ISC-EB vs. ISC-ISC) are with unpaired *t* test, which also show significant differences. Data are from supplementary table associated with Li et al.’s *SI Appendix*, figure S8 and are represented as mean ± SD.

To determine whether a histone is asymmetrically inherited, the proper statistics should assess deviation from a 1:1 ratio (log_2_ = 0). Reanalysis reveals H3.1 asymmetric distribution in ISC-EB and symmetric distribution in ISC-ISC. Biologically meaningful comparison includes comparing the same histone across different division modes. These reanalyses indicate division mode-specific features (Fig. 1) and overall consistency with our work (2). In Li et al., statistical comparisons were made between old and new histones within the same division mode. To better visualize their relationship, two-dimensional plots can display both inheritance patterns and directionality [(2)’s figure 3, (3)’s *SI Appendix*, figure S3], considering that both may exhibit the same directionality, as previously reported for nucleosomal density asymmetries (4).

**Fig. 2.**
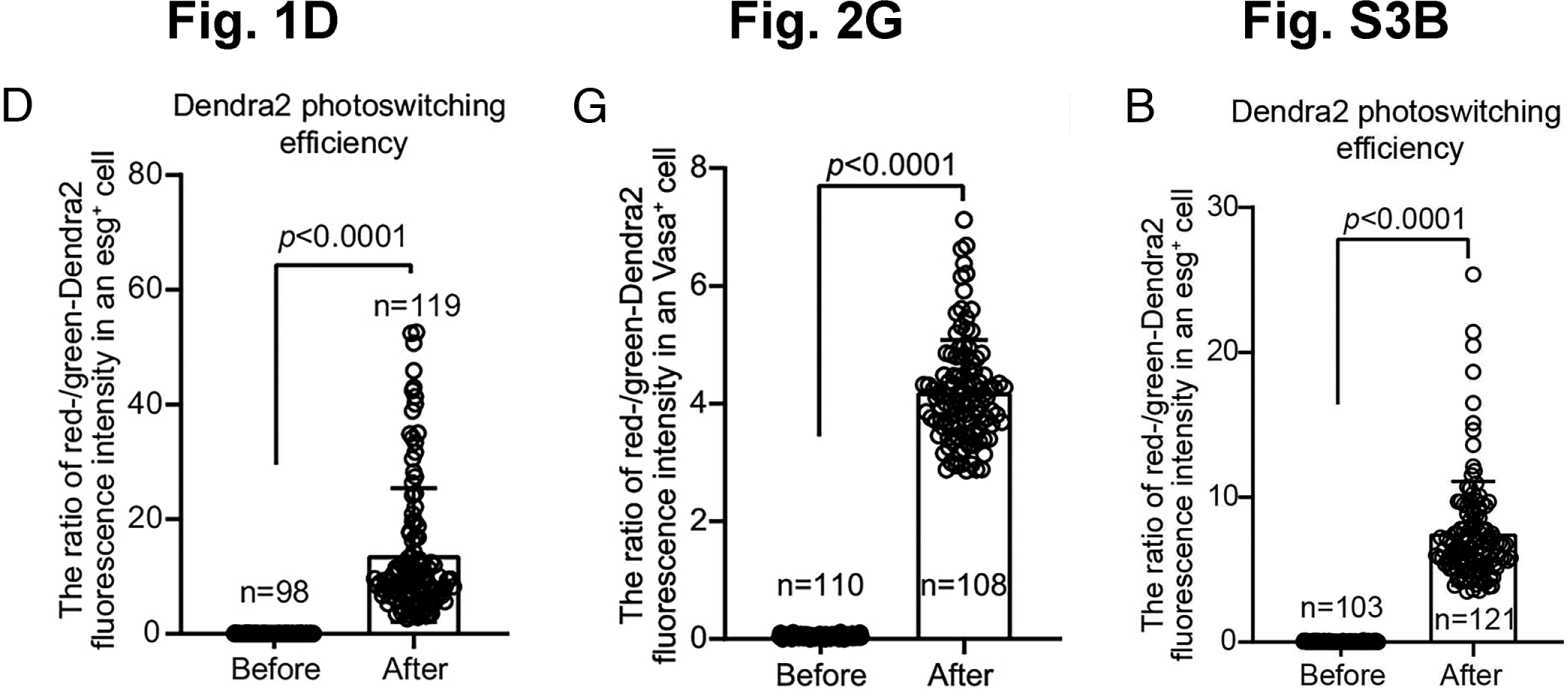
Unreliable measurement of Dendra2 photoconversion efficiency. The authors estimated photoconversion efficiency using “the ratio of red-/green-Dendra2 fluorescence intensity” (Li et al.’s figures 1*D* and 2*G* and *SI Appendix*, figure S3*B* with widely varying *Y*-axis scales), which we assert is an unreliable metric because the two fluorescence channels differ in intensity and detection sensitivity. Moreover, before photoconversion, all values are zero due to the absence of red-Dendra2 signal. After photoconversion, any detectable red-Dendra2 signal yields a nonzero value regardless of efficiency. We argue that neither the magnitude of the measurement nor the associated *P*-values meaningfully reflects photoconversion efficiency. In our view, a more appropriate calculation is [Green-Dendra2 (before conversion) − Green-Dendra2 (after conversion)]/Green-Dendra2 (before conversion), which quantifies the percentage of Green-Dendra2 that was photoconverted, as previously described (5, 6). Because unconverted old Green-Dendra2 can be misinterpreted as newly synthesized histones, we argue that it is difficult to draw confident conclusions from all these photoconversion experiments.

Li et al. generated several Dendra2-tagged lines to distinguish old and new histones through photoconversion. However, photoconversion efficiency was measured using a metric we regard as unreliable (Fig. 2), leading us to question the validity of their conclusions.

An important technical consideration is that fixed Dendra2 remains photoconvertible. Therefore, the UV channel should be avoided or used last. The presence of BFP-like signals (Li et al.’s figures 1–4 and *SI Appendix*, figures S3–S6) and the lack of imaging acquisition order information raise concerns that photoconversion may still occur during imaging. Additional technical issues may further limit data interpretation. Multiple images show bleed through: Somatic hub signals appear in Vasa^+^ germline channel (Li et al.’s figures 2 *D* and *E*, 3*C*, and 4*F* and *SI Appendix*, figures S6*G*, S10*A*, and S10*C*), and PH3 signals may bleed into other channels, potentially compromising quantitative accuracy. The imaging data require further clarification.

Canonical H3.1 is mainly incorporated during S-phase, thus old vs. new histone segregation can only be reliably assessed in cells completing a S-phase. If the switch occurs in G2-phase, the subsequent mitosis is inappropriate for evaluating these [(7)’s figure 3*K*]. Although they allowed a relatively long recovery, both UV-exposure and heat-shock can induce cell-cycle arrest. Additional controls are needed: In our tag-switch system, we identify mitotic cells with robust new canonical H3.1 signals. However, inefficient photoconversion could confound this control.

Furthermore, analyses of postmitotic pairs should include appropriate controls, because histone patterns in cycling cells may not faithfully reflect inheritance from the preceding stem cell division. Since ISCs can reenter S-phase, an EdU pulse would allow exclusion of EdU^+^ ISC-EB pairs from analysis [(2)’s *Method*]. Such effects may account for the observed reduction of H3.1-GFP in the ISC of ISC-EB pairs (Fig. 1), whereas our results indicate the opposite (2). Finally, an ISC-specific driver, such as *esg-Gal4; Su(H)-Gal80*, can restrict EB-expression when using the *UAS*-tag-switch system (2).

We acknowledge the limitations of the transgene design and the challenges of applying it to different systems. We developed complementary approaches, such as live-cell imaging (4, 8) and antibody-based detection (9), as discussed in previous publications, including a review (10).
